# Bibliography

**Published:** 1897-08

**Authors:** 


### Bibliography.

A Practical Treatise on Mechanical Dentistry. By Joseph Richardson, M.D., D.D.S., Late Professor . . . in the Indiana Dental College, etc., etc. Seventh edition, revised, enlarged, and edited by George W. Warren, D.D.S. With six hundred and ninety-one illustrations, many of which are from new and original drawings. P. Blakiston, Son & Co., Philadelphia, 1897.

The seventh edition of Richardson's Mechanical Dentistry expresses in a few words the demand for this text-book, which has for so long a period been recognized as an authority upon that important branch of dentistry. It has become the habit of a certain class of mind to cavil at the mechanical branch of dental work as something that a professional man should relegate to those who make of it a specialty. It is safe, however, to assume that the dentist who fails to familiarize himself with the technique of this part of his work is not a dentist in the best sense of the word, for mere theory never did or will make a man an acceptable practitioner. It is a satisfaction, therefore, to find that this standard work continues, in the hands of the present editor, fully abreast of all the improvements made, and this is evident in the elaboration of all the chapters; in fact, there is an entire revision of the work, making it, practically, not a new edition, but a new work.

For one man to do this, in the complex relations which mechanical dentistry has assumed in the last decade, means an ability of no ordinary character, and Dr. Warren should be congratulated that he has succeeded so well in overcoming all the difficulties insuperably connected with a work of this kind.

The tendency of the present period is to make text-books through collaboration of many minds. This is a recognition of the division of prosthetic dentistry into several specialties. This has its good and its bad expression. Good, in that it is likely to give a greater variety of thought, as well as a direct system of individual practice; and bad, as it tends to giving the book an encyclopædic character, thus losing that coherence of thought so important in text-books. On the other hand, the individual production means a loss of system, for it is next to impossible for one person to gather all the ideas of individuals into a condensed form and give the original thoughts full justice. The result is apt to be confusing to the beginner, however valuable it may be, as a means of reference, to the experienced operator. This difficulty is nowhere more apparent than in the multiplication of methods and so-called systems of crown- and bridge-work. If these were all reduced to compact and orderly arrangement, it would lessen the bulk of the volume and add much to it as a valuable aid in teaching.

The editor has made notable improvements in many of the chapters, especially in the important matter of soldering, one of the most difficult operations the student is forced to meet and conquer.

In a book of this kind criticism, as to many minor defects, would be out of place. The general make-up and careful discrimination in the selection of methods is worthy of highest praise. In future editions it would add greatly to the value of the book if detailed descriptions were employed. In illustration of this need reference may be made to the preparation of lower plates. There is probably no more difficult piece of work for the beginner than this, and all the steps in the process should be detailed, especially in the matter of soldering two plates together.

The critical examination of this book carries with it a feeling of satisfaction that the year 1897 has given to dentistry two text-books on mechanical work, each worthy of high commendation, and evidencing a growing tendency to cherish the practical side of our calling as worthy to extend upon parallel lines with that regarded as more strictly professional. It is, therefore, gratifying to feel that Richardson's Mechanical Dentistry will retain its place as a text-book, at all times valuable and indispensable for reference for both practitioners and students.

Some Methods and Appliances in Operative and Mechanical Dentistry. By R. P. Lennox. With illustrations. Claudius Ash & Sons, Limited, London, 1897.

In this little book of one hundred and twenty pages, it is safe to assert, will be found more original and suggestive paragraphs than will be discovered, in similar space, in more pretentious volumes devoted to mechanical dentistry. The author, R. P. Lennox, is well known in dental circles in England for his many original ideas. These he has compiled in book form, and will present a novel character to those familiar with processes upon this side of the water, and must impress the mind with their simplicity and apparent effectiveness.

The author says in his preface, "An attempt has been made to reply once for all to questions put to the author from time to time with regard to papers he has read before the British Dental Association and its branches. . . . Advantage has also been taken of the opportunity afforded by the carrying out of these purposes to add a few hints on matters not found in the text-books, and, further, to give an account of those of the author's methods of working which differ from the methods generally to be found there."

In accordance with the custom in Europe, much space is given to the use of springs, long since relegated to the unknown in this country. This latter fact is, perhaps, to be regretted, for cases occur in practice where these could be used to advantage; and this is practically the author's position, for he says, "In the majority of cases well-made plates need no special contrivance to keep them in position." His suggestions upon taking the "bite" and flasking are excellent, and might generally be adopted with advantage.

The use of Ash & Son's teeth bring into prominence some methods certainly novel to the average American dentist. His illustration of a cutting instrument to *shorten teeth*, where required, is a process, it is imagined, not even thought of here, much less attempted with the ordinary porcelain teeth in use.

The chapter on fusible metal is replete with valuable suggestions and well worthy of quoting entire, but without the profusely illustrated pages would not be clearly understood. The same may be said of the chapter on matrices.

It is not necessary that this original production should be followed in this review from preface to appendix. Every page indicates the mechanic and thinker, and one capable of describing his methods and with the courage to follow them out, whether in agreement with established usage or otherwise. American operators might do well to study the methods herein detailed, for while they will naturally not agree with some of them, they cannot fail to secure ideas of value and of great assistance in facilitating operations.

